# Tetrahydrofuran Cembranoids from the Cultured Soft Coral *Lobophytum crassum*

**DOI:** 10.3390/md9122526

**Published:** 2011-12-07

**Authors:** Nai-Lun Lee, Jui-Hsin Su

**Affiliations:** 1 National Museum of Marine Biology & Aquarium, Pingtung 944, Taiwan; Email: love4brat1@yahoo.com.tw; 2 Graduate Institute of Marine Biotechnology, National Dong Hwa University, Pingtung 944, Taiwan

**Keywords:** soft coral, *Lobophytum crassum*, cembranoids

## Abstract

Three new cembranoids, culobophylins A–C (**1**–**3**), along with two known compounds (**4** and **5**) were isolated from the cultured soft coral *Lobophytum crassum*. The structures of these compounds were elucidated on the basis of their spectroscopic data and comparison of the NMR data with those of known analogues. Among these metabolites, **2** is rarely found in cembranoids possessing an isopropyl moiety with an epoxide group. Compound **1** exhibited significant cytotoxic activity against HL60 and DLD-1 cancer cell lines.

## 1. Introduction

**Figure 1:**
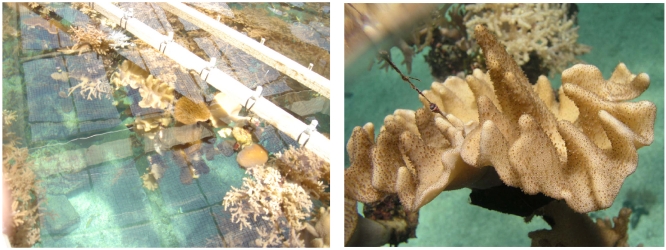
Soft coral *Lobophytum crassum*.

In the investigation of secondary metabolites from marine invertebrates, several terpenoid metabolites have been isolated from cultured octocorals *Erythropodium* [1], *Klyxum simplex* [2,3,4], *Sinularia flexibilis* [5], *Sarcophyton trocheliophorum* [6], *Briareum excavatum* [7,8,9,10,11,12,13,14,15] and *Briareum* sp. [16]. Some of these metabolites have been found to possess several kinds of biological activities, such as cytotoxic [2,4,5,8,16] and anti-inflammatory activities [3,4,11,12,13,14]. The current chemical investigation of cultured octocoral *Lobophytum crassum* (Figure 1) led to the discovery of three new cembranoids, culobophylins A–C (**1**–**3**), and two known compounds lobophylin B (**4**), and lobophylin A (**5**) [17]. The structures of **1**–**5** were established by detailed spectroscopic analysis, including extensive examination of 2D NMR (^1^H–^1^H COSY, HMQC and HMBC) correlations. The cytotoxicity of metabolites **1**–**5** against human promyelocytic leukemia (HL60), human breast carcinoma (MDA-MB-231) and human colon adenocarcinoma (HCT-116 and DLD-1) cell lines was studied, and the ability of **1**–**5** to inhibit the expression of the pro-inflammatory iNOS (inducible nitric oxide synthase) and COX-2 (cyclooxygenase-2) proteins in lipopolysaccharide (LPS)-stimulated RAW264.7 macrophage cells was also evaluated.

## 2. Results and Discussion

**Chart 1:**
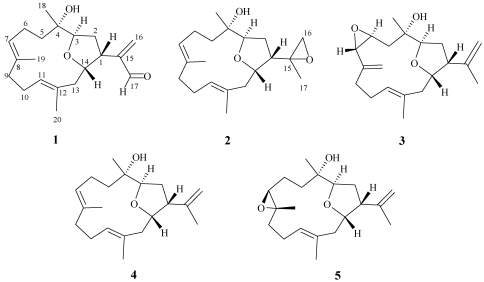
Structures of metabolites **1**–**5**.

The EtOAc extract of the freeze-dried specimen was fractionated by silica gel column chromatography and the eluted fractions were further separated utilizing normal phase HPLC to yield metabolites **1**–**5** (Chart 1). 

**Figure 2:**
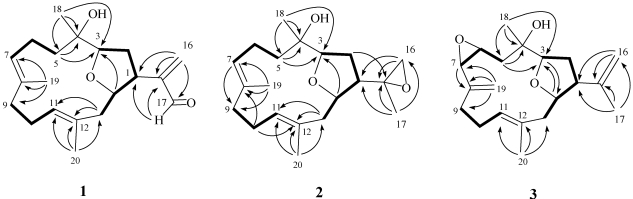
Selected ^1^H−^1^H COSY (▬) and HMBC (→) correlations of **1**–**3**.

**Figure 3:**
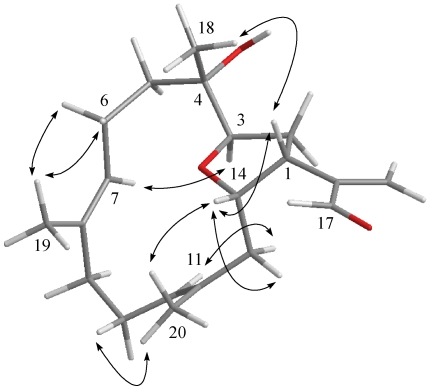
Computer-generated model of **1** using MM2 force field calculations and key NOE correlations.

The new metabolite culobophylin A (**1**) had a molecular formula of C_20_H_30_O_3_, which was determined by HRESIMS and NMR spectroscopic data. The IR spectrum of **1** showed absorption bands at 3458 and 1694 cm^−1^, suggesting the presence of hydroxy and carbonyl groups. The ^13^C NMR data of **1** showed the presence of 20 carbons (Table 1): three methyls, six sp^3^ methylenes, one sp^2^ methylene, three sp^3^ methines (including two oxygenated carbons at δ 76.6 and 75.6), two sp^2^ methines, and one sp^3^ quaternary carbon. The remaining three signals appearing in the lower field region of the spectrum are due to the quaternary carbons of three olefinic carbons (δ 148.2, 132.9 and 131.8) and one aldehyde carbonyl (δ 194.7). From the ^1^H NMR (Table 1) spectrum of **1**, the presence of one aldehyde proton resonating as a singlet at δ_H_ 9.56 was observed. Moreover, the ^1^H NMR data revealed the presence of two olefinic methylene protons (δ 6.33, *J* = 1.5 Hz; 6.14, d, *J* = 1.5 Hz) and two olefinic methine protons (δ 5.18, dd, *J* = 5.0, 5.0 Hz; 4.84, d, *J* = 7.5 Hz). Furthermore, two oxygenated methines (δ 4.66, ddd, *J* = 11.0, 6.0, 5.0 Hz; 3.96, dd, *J* =9.5, 4.0 Hz) and three methyls (δ 1.61, s; 1.56, s; 1.10, s) were also designated from the ^1^H NMR signals. The planar structure and all of the ^1^H and ^13^C chemical shifts of **1** were elucidated by 2D NMR spectroscopic analysis, in particular ^1^H–^1^H COSY and HMBC experiments (Figure 2). From the ^1^H–^1^H COSY correlations (Figure 2), it was possible to establish three partial structures of consecutive proton spin systems extending from H_2_-5 to H-7; H-9 to H-11; H_2_-13 to H-3. The following key HMBC correlations permitted connection of the carbon skeleton: H_2_-5 to C-3 and C-4; H_2_-13 to C-11 and C-12; H_2_-16 to C-1, C-15 and C-17; H-17 to C-1 and C-15; H_3_-18 to C-3, C-4 and C-5; H_3_-19 to C-7, C-8 and C-9; and H_3_-20 to C-11, C-12 and C-13. Thus, **1** was found to possess three double bonds at C-7/C-8, C-11/C-12 and C-15/C-16 and an aldehyde group at C-15. Furthermore, the HMBC cross-peak from H-14 to C-3 suggested that C-3 and C-14 were linked through an oxygen to form a tetrahydrofuran ring. The relative configuration of **1** elucidated mainly from the NOESY spectrum was compatible with that of **1** offered by using the MM2 force field calculations which suggested the most stable conformations as shown in Figure 3. In the NOESY spectrum, both H_3_-18 and H-14 showed NOEs with H-1 but not with H-3. Thus, assuming the *β*-orientation of H-1, H_3_-18 and H-14 should be positioned on the *β* face. Moreover, H-3 should be positioned on the α face. Also, the NOE correlations of H_3_-19 with H_2_-6 but not with H-7 and H_3_-20, with H-10a (δ 2.33) but not with H-11, indicated the *E* configuration of the double bonds between C-7/C-8 and C-11/C-12. Furthermore, the relative stereochemistry of **1** was mostly confirmed to be the same as that of **4** by comparison of the proton chemical shifts and coupling constants [17]. On the basis of the above findings and other detailed NOE correlations, the structure of **1** was established unambiguously.

**Table 1:** ^1^H and ^13^C NMR data for **1**–**3**.

Position	1		2		3	
	δ_H_ (*J* in Hz) ^a^	δ_c_ (mult.) ^b^	δ_H_ (*J* in Hz) ^a^	δ_c_ (mult.) ^b^	δ_H_ (*J* in Hz) ^a^	δ_c_ (mult.) ^b^
1	3.12 dt (10.0, 8.5) *^c^*	41.1 (CH) *^d^*	2.53 m	46.1 (CH)	2.75 dt (6.0, 5.5)	49.8 (CH)
2	2.12 m; 2.04 m	27.3 (CH_2_)	1.70 m	24.7 (CH_2_)	2.08 m	31.3 (CH_2_)
3	3.96 dd (9.5, 4.0)	76.6 (CH)	3.91 dd (9.5, 4.0)	77.3 (CH)	4.13 dd (7.5, 7.5)	82.5 (CH)
4		74.2 (C)		74.2 (C)		74.4 (C)
5	2.00 m; 1.54 m	38.7 (CH_2_)	1.94 m; 1.52 m	38.6 (CH_2_)	2.18 dd (13.5, 3.5); 1.55 m	46.2 (CH_2_)
6	2.22 m; 2.06 m	21.4 (CH_2_)	2.17 m; 2.04 m	21.4 (CH_2_)	3.46 ddd (8.5, 2.5, 2.0)	54.5 (CH)
7	5.18 dd (5.0, 5.0)	126.4 (CH)	5.15 dd (5.5, 5.5)	126.2 (CH)	3.10 d (2.0)	61.6 (CH)
8		132.9 (C)		133.1 (C)		146.5 (C)
9	2.14 m; 1.98 m	38.1 (CH_2_)	2.15 m; 2.02 m	38.1 (CH_2_)	2.14 m; 1.82 m	27.9 (CH_2_)
10	2.33 m; 2.04 m	24.4 (CH_2_)	2.35 m; 2.04 m	24.4 (CH_2_)	2.27 m	28.6 (CH_2_)
11	4.84 d (7.5)	127.2 (CH)	4.92 d (8.5)	127.5 (CH)	5.16 dd (7.5, 7.5)	125.0 (CH)
12		131.8 (C)		131.5 (C)		132.5 (C)
13	1.71 m; 1.51 m	40.1 (CH_2_)	2.28 m; 2.08 m	39.9 (CH_2_)	1.92 d (6.5)	39.4 (CH_2_)
14	4.66 ddd (11.0, 6.0, 5.0)	75.6 (CH)	4.42 ddd (11.0, 5.5, 5.5)	76.8 (CH)	4.05 dd (7.0, 7.0)	79.0 (CH)
15		148.2 (C)		54.2 (C)		144.4 (C)
16	6.33 d (1.5); 6.14 d (1.5)	134.9 (CH_2_)	2.51 d (4.5); 2.43 d (5.0)	50.8 (CH_2_)	4.83 s; 4.73 s	112.3 (CH_2_)
17	9.56 s	194.7 (CH)	1.37 s	22.1 (CH_3_)	1.77 s	22.1 (CH_3_)
18	1.10 s	23.1 (CH_3_)	1.07 s	22.9 (CH_3_)	1.15 s	21.9 (CH_3_)
19	1.56 s	16.4 (CH_3_)	1.58 s	16.4 (CH_3_)	5.28 s; 5.12 s	115.0 (CH_2_)
20	1.61 s	15.3 (CH_3_)	1.67 s	15.3 (CH_3_)	1.62 s	17.1 (CH_3_)

*^a^* 500 MHz in CDCl_3_; *^b^* 125 MHz in CDCl_3_; *^c^* *J* values (Hz) are given in parentheses; *^d^* Numbers of attached protons were deduced by DEPT experiments.

Culobophylin B (**2**) was isolated as a colorless oil with the molecular formula C_20_H_32_O_3_, which possesses five degrees of unsaturation, as indicated by HRESIMS (*m*/*z* 343.2251, [M + Na]^+^) and NMR spectroscopic data (Table 1). In addition, ^1^H and ^13^C NMR spectroscopic data (Table 1) of **2** showed the structural unit of a 3,14-oxa-bridged tetrahydrofuran. By comparison of the NMR data of **2** with that of **4**, it was found that the ^1^H and ^13^C NMR data of **2** were very similar to those of **4** [17]. However, the ^1^H and ^13^C NMR spectroscopic data revealed that the signals corresponding to one 1,1-disubstituted carbon–carbon double bond in **4** were not present and were replaced by one 1,1-disubstituted epoxide in **2** (δ_H_ 2.51, 1H, d, *J* = 4.5 Hz and δ_H_ 2.43, 1H, d, *J* = 5.0 Hz; δ_C_ 54.2, C and δ_C_ 50.8 CH_2_) (Table 1). ^1^H–^1^H COSY and HMBC (Figure 2) further revealed that **2** possesses one 1,1-disubstituted epoxide at C-15. On the basis of the above observations, and with the assistance of additional 2D NMR (^1^H–^1^H COSY and HMBC) correlations, it was possible to establish the planar structure of **2**, as illustrated in Figure 2. The relative stereochemistries of all stereocenters except C-15 of **2** were confirmed to be the same as those of **1** and **4** by comparison of the proton shifts, coupling constants, and NOE correlations of **2** with those of **1** and **4**.

**Figure 4:**
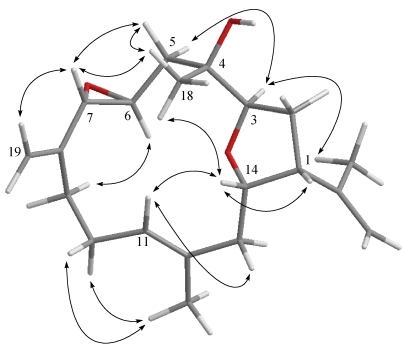
Computer-generated model of **3** using MM2 force field calculations and key NOE correlations.

Culobophylin C (**3**) was obtained as a colorless oil and showed a [M + Na]^+^ ion peak in the HRESIMS spectrum corresponding to the molecular formula C_20_H_30_O_3_, the same as that of **2**. IR absorptions were observed at 3425 cm^−1^, suggesting the presence of a hydroxy group in **3**. The ^13^C NMR spectrum of **3** showed twenty signals accounting for three methyls, five sp^3^ methylenes, two sp^2^ methylenes, five sp^3^ methines , one sp^2^ methine and four quaternary carbons (including one oxygenated carbon at δ 74.4 and three olefinic carbons with resonances at δ 146.5, 144.4 and 132.5). The ^1^H NMR data revealed the presence of four olefinic methylene protons (δ 5.28, 5.12, 4.83 and 4.73, each a singlet). Two proton signals at δ 3.46 ddd (1H, 8.5, 2.5, 2.0) and 3.10 (1H, d, *J* = 2.0 Hz) correlated with two carbon signals at δ 54.5 and 61.6 and in the HMQC spectrum of **3** were attributed to the proton of one 1,2-disubstituted epoxide. The planar structure and all of the ^1^H and ^13^C chemical shifts of **3** were elucidated by 2D NMR spectroscopic analysis, in particular ^1^H–^1^H COSY and HMBC experiments (Figure 2). Thus, **3** was found to possess three double bonds at C-8/C-19, C-11/C-12 and C-15/C-16, one hydroxy group at C-4, one 1,2-disubstituted epoxide at C-6/C-7, and an oxa-bridged ether linkage at C-3/C-14. The relative configurations of the five chiral centers at C-3, C-4, C-6, C-7 and C-14 in **3** were elucidated by detailed NOE analysis, as shown in Figure 4. In these experiments, it was found that H_3_-18 showed NOE interactions with H-14 and H-7. Thus, assuming the *β*-orientation of H_3_-18, H-7 and H-14 should be positioned on the *β* face. The NOE correlation observed between H-14 and H-1 also reflected the *β*-orientation of H-1. Furthermore, the NOESY spectrum showed NOE interaction of H_3_-20 with H-10, but not with H-11, revealing the *E* geometry of the C-11/C-12 double bond. On the basis of these results and other detailed NOE correlations, the structure of **3** was established unambiguously.

**Figure 5:**
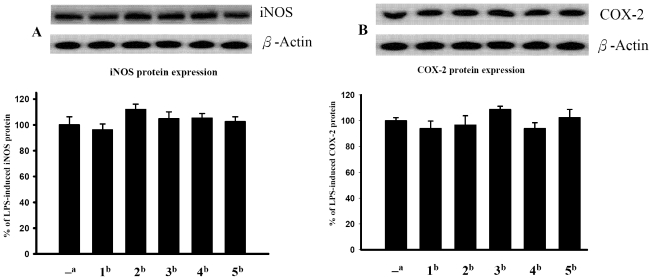
Effect of compounds **1**–**5** at 10 μM on the expression of iNOS and COX-2 proteins of RAW264.7 macrophage cells examined by immunoblot analysis. (**A**) Immunoblots of iNOS and β-actin; (**B**) immunoblots of COX-2 and β-actin. Values represent mean ± SEM (*n* = 6). The relative intensity of the LPS-only-stimulated group was taken as 100%. * Significantly different from the LPS-only-stimulated group (* *P* < 0.05). *^a^* Stimulated with LPS; *^b^* stimulated with LPS in the presence of **1**–**5**.

The cytotoxicities of compounds **1**–**5** against HL60, MDA-MB-231, DLD-1 and HCT-116 cancer cells are shown in Table 2. The results show that compound **1**, the most potent of compounds **1**–**5**, exhibited cytotoxicity against the HL60, MDA-MB-231, DLD-1 and HCT-116 cancer cell lines with IC_50_s of 3.0, 16.8, 4.6 and 16.3 μg/mL, respectively. Furthermore, compound **2** exhibited moderate to weak cytotoxic activity against HL60, DLD-1 and HCT-116 cancer cell lines (the IC_50_ values were 6.8, 16.2 and 16.7 μg/mL for HL60, DLD-1 and HCT-116, respectively). The other tested compounds were not cytotoxic (IC_50_ > 20 μg/mL) toward the above four cancer cell lines. The *in vitro* anti-inflammatory effects of **1**–**5** were also tested. Furthermore, the anti-inflammatory activity of **1**–**5** against the accumulation of pro-inflammatory iNOS and COX-2 proteins in RAW264.7 macrophage cells stimulated with LPS was evaluated using immunoblot analysis. At a concentration of 10 µM, compounds **1**–**5** did not inhibit COX-2 and iNOS proteins expression relative to the control cells stimulated with LPS only (Figure 5). 

**Table 2:** Cytotoxicity (IC_50_ μg/mL) of compounds **1**–**5**.

Compound	Cell Lines			HCT-116
	HL60	MDA-MB-231	DLD-1	
**1**	3	16.8	4.6	16.3
**2**	6.8	– ^a^	16.2	16.7
**3**	– ^a^	– ^a^	– ^a^	– ^a^
**4**	– ^a^	– ^a^	– ^a^	– ^a^
**5**	– ^a^	– ^a^	– ^a^	– ^a^
Doxorubicin C	0.05	6.3	5.7	0.5

^a^ IC_50_ > 20 μg/mL.

## 3. Experimental Section

### 3.1. General Experimental Procedures

Optical rotation values were measured using a Jasco P-1010 digital polarimeter. IR spectra were recorded on a Varian Digilab FTS 1000 Fourier transform infrared spectrophotometer. The NMR spectra were recorded on a Varian Unity INOVA 500 FT-NMR instrument at 500 MHz for ^1^H NMR and 125 MHz for ^13^C NMR, respectively, in CDCl_3_. ESIMS and HRESIMS data were recorded with a Bruker APEX II mass spectrometer. Gravity column chromatography was performed on silica gel (230–400 mesh, Merck). TLC was carried out on precoated Kieselgel 60 F254 (0.2 mm, Merck) and spots were visualized by spraying with 10% H_2_SO_4_ solution followed by heating. High-performance liquid chromatography was performed using a system comprised of a Hitachi L-7100 pump and a Rheodyne 7725 injection port. A preparative normal phase column (250 × 21.2 mm, 5 μm) was used for HPLC.

### 3.2. Animal Material

Specimens of the soft coral *Lobophytum crassum* were collected off the coast of Pingtung, southern Taiwan, and transplanted to a 120-ton cultivating tank equipped with a flow-through sea water system in July 2003. The cultured soft coral was harvested in December 2010. A voucher specimen (specimen no. 2010CSC-1) was deposited in the National Museum of Marine Biology and Aquarium, Taiwan.

### 3.3. Extraction and Separation

The frozen bodies of soft coral (5.0 kg, fresh wt.) were collected and freeze-dried. The freeze-dried material was minced and extracted exhaustively with EtOAc (5 × 2 L). The organic extract was evaporated to yield a residue (60.5 g), which was fractionated by open column chromatography on silica gel using *n*-hexane–EtOAc and EtOAc–acetone mixtures of increasing polarity to yield 15 fractions. Fraction 6, eluting with *n*-hexane-EtOAc (15:1), was further separated by silica gel column chromatography with gradient elution (*n*-hexane-EtOAc, 15:1 to 10:1) to yield five subfractions (6A–6E). Subfraction 6C was subjected to normal phase HPLC (*n*-hexane-EtOAc, 15:1) to obtain compound **4** (3.0 mg). Fraction 8, eluting with *n*-hexane-EtOAc (5:1), was further separated by silica gel column chromatography with gradient elution (*n*-hexane-EtOAc, 5:1 to 2:1) to give six subfractions (8A–8F). Subfraction 8B was separated by normal phase HPLC using *n*-hexane-EtOAc (5:1) to afford **5** (2.5 mg). In the same manner, compound **3** (2.0 mg) was obtained from subfraction 8 D normal phase HPLC (*n*-hexane-EtOAc, 3:1). Fraction 9, eluting with *n*-hexane-EtOAc (3:1), was further separated by silica gel column chromatography with gradient elution (*n*-hexane-EtOAc, 3:1 to 1:1) to yield five subfractions (9A–9E). Subfraction 9C was further purified by was subjected to normal phase HPLC (*n*-hexane-EtOAc, 2:1) to obtain compounds **1**(1.2 mg) and **2** (3.0 mg).

Culobophylin A (**1**): colorless oil; 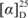 = −50 (*c* 0.1, CHCl_3_); IR (neat) ν_max_ 3458, 2924, 2853, 1694, 1458 and 1377 cm^−^^1^; ^1^H and ^13^C NMR data, see Table 1; ESIMS *m*/*z* 341 [100, (M + Na)^+^]; HRESIMS *m*/*z* 341.2091 (calcd. for C_20_H_30_O_3_Na, 341.2093).

Culobophylin B (**2**): colorless oil; 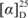 = −24 (*c* 0.3, CHCl_3_); IR (neat) ν_max_ 3499, 2925, 2853, 1457, 1382 and 1264 cm^−^^1^; ^1^H and ^13^C NMR data, see Table 1; ESIMS *m*/*z* 343 [100, (M + Na)^+^]; HRESIMS *m*/*z* 343.2251 (calcd. for C_20_H_32_O_3_Na, 341.2249).

Culobophylin C (**3**): colorless oil; 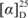 = −83 (*c* 0.3, CHCl_3_); IR (neat) ν_max_ 3425, 2923, 1638, and1459 cm^−^^1^, ^1^H and ^13^C NMR data, see Table 1; ESIMS *m*/*z* 341 [100, (M + Na)^+^]; HRESIMS *m*/*z* 341.2095 (calcd. for C_20_H_30_O_3_Na, 341.2093).

Lobophylin B (**4**): colorless oil; 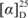 = −30 (*c* 0.5, CHCl_3_); 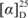 [lit. = −35 (*c* 0.3, CHCl_3_) [17]].

Lobophylin A (**5**): colorless oil; 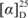 = −45 (*c* 0.3, CHCl_3_); [lit. 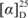 = −39 (*c* 0.3, CHCl_3_) [17]].

### 3.4. Cytotoxicity Testing

Cell lines were purchased from the American Type Culture Collection (ATCC). Cytotoxicity assays of compounds **1**–**5** were performed using the MTT [3-(4,5-dimethylthiazol-2-yl)-2,5-diphenyltetrazolium bromide] colorimetric method [18,19]. 

### 3.5. *In Vitro* Anti-Inflammatory Assay

Macrophage (RAW264.7) cell line was purchased from ATCC. *In vitro* anti-inflammatory activities of compounds **1**-**5** were measured by examining the inhibition of lipopolysaccharide (LPS) induced upregulation of iNOS (inducible nitric oxide synthetase) and COX-2 (cyclooxygenase-2) proteins in macrophages cells using western blotting analysis [20,21].

### 3.6. Molecular Mechanics Calculations

Implementation of the MM2 force filed in Chem3D Pro software [22], was used to calculate the molecular models.

## 4. Conclusions

In previous reports, several 3,14-ether linkage-related cembranoids were identified from the marine soft corals *Sinularia gibberosa* [23,24], *Sarcophyton infundibuliforme* [25] and *Lobophytum* sp. [17]. Among these compounds, only one (3,14-epoxy-1(*E*),7(*E*),11(*E*)-cembratrien-4,15-diol) has been found to possess moderate cytotoxicity toward three cancer cells (A-549, HT-29 and P-388) [24]. In the present study, only compound **1** exhibited significant cytotoxicity against the growth of HL60 and DLD-1 cancer cell lines. According to the structures of **1**–**5**, it seems that the aldehyde group in compound **1** is critical for the cytotoxic activity of metabolites **1**–**5**. It is worth noting that metabolite **2** is rarely found in cembranoids possessing an isopropyl moiety with an epoxide group [26].

## Supplementary Files

Supplementary File 1: PDF-Document (PDF, 1263 KB)
