# Supplementary material for: Tetrahydrofuran Cembranoids from the Cultured Soft Coral Lobophytum crassum
Source: Mar Drugs. 2011 Dec 7;9(12):2526–36. doi: 10.3390/md9122526 (PMC3280583; doi:10.3390/md9122526)
Supplement: Supplementary File 1: — PDF-Document (PDF, 1263 KB) [file marinedrugs-09-02526-s001.pdf]

Supporting Information

Tetrahydrofuran Cembranoids from the Cultured Soft Coral *Lobophytum crassum*

Nai-Lun Lee and Jui-Hsin Su*

Table of Contents

S1. Table of Contents

S2. ^{1}H NMR spectrum of **1** in $CDCl_3$ at 500 MHz.

S3. ^{13}C NMR spectrum of **1** in $CDCl_3$ at 125 MHz.

S4. ^{1}H NMR spectrum of **2** in $CDCl_3$ at 500 MHz.

S5. ^{13}C NMR spectrum of **2** in $CDCl_3$ at 125 MHz.

S6. ^{1}H NMR spectrum of **3** in $CDCl_3$ at 500 MHz.

S7. ^{13}C NMR spectrum of **3** in $CDCl_3$ at 125 MHz.

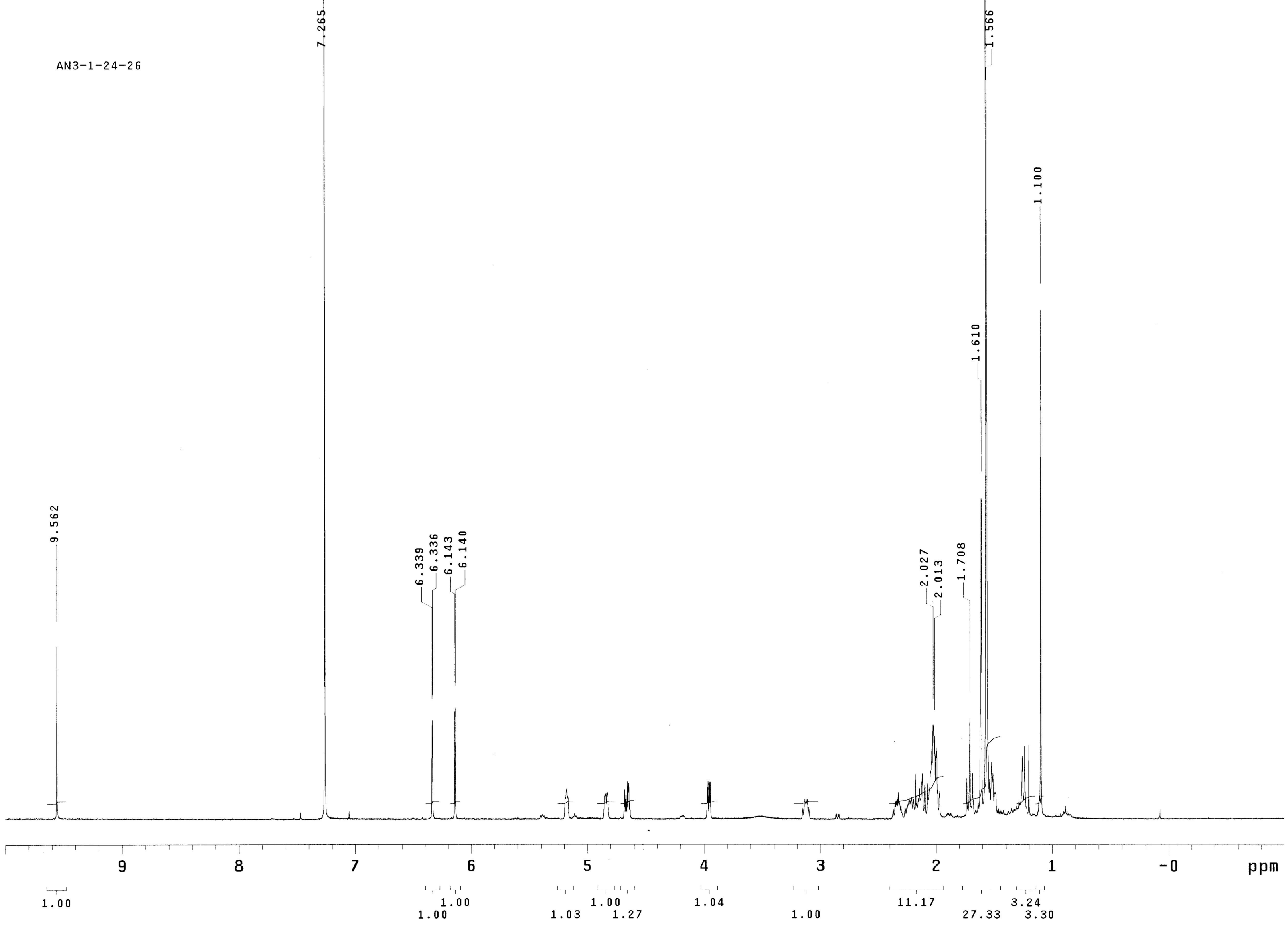

S2. ^{1}H NMR spectrum of **1** in $CDCl_3$ at 500 MHz.

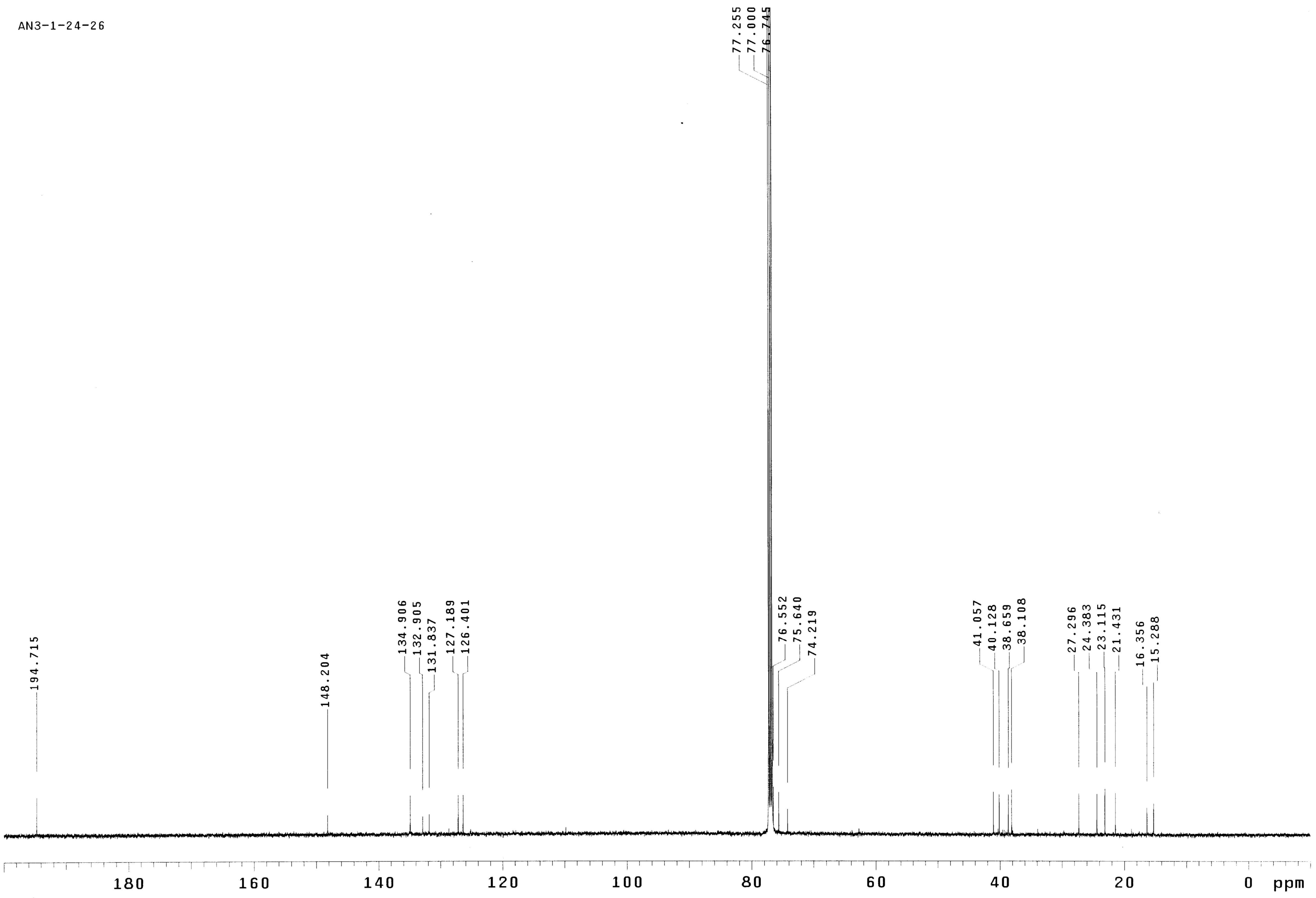

S3. ^{13}C NMR spectrum of **1** in $CDCl_3$ at 125 MHz.

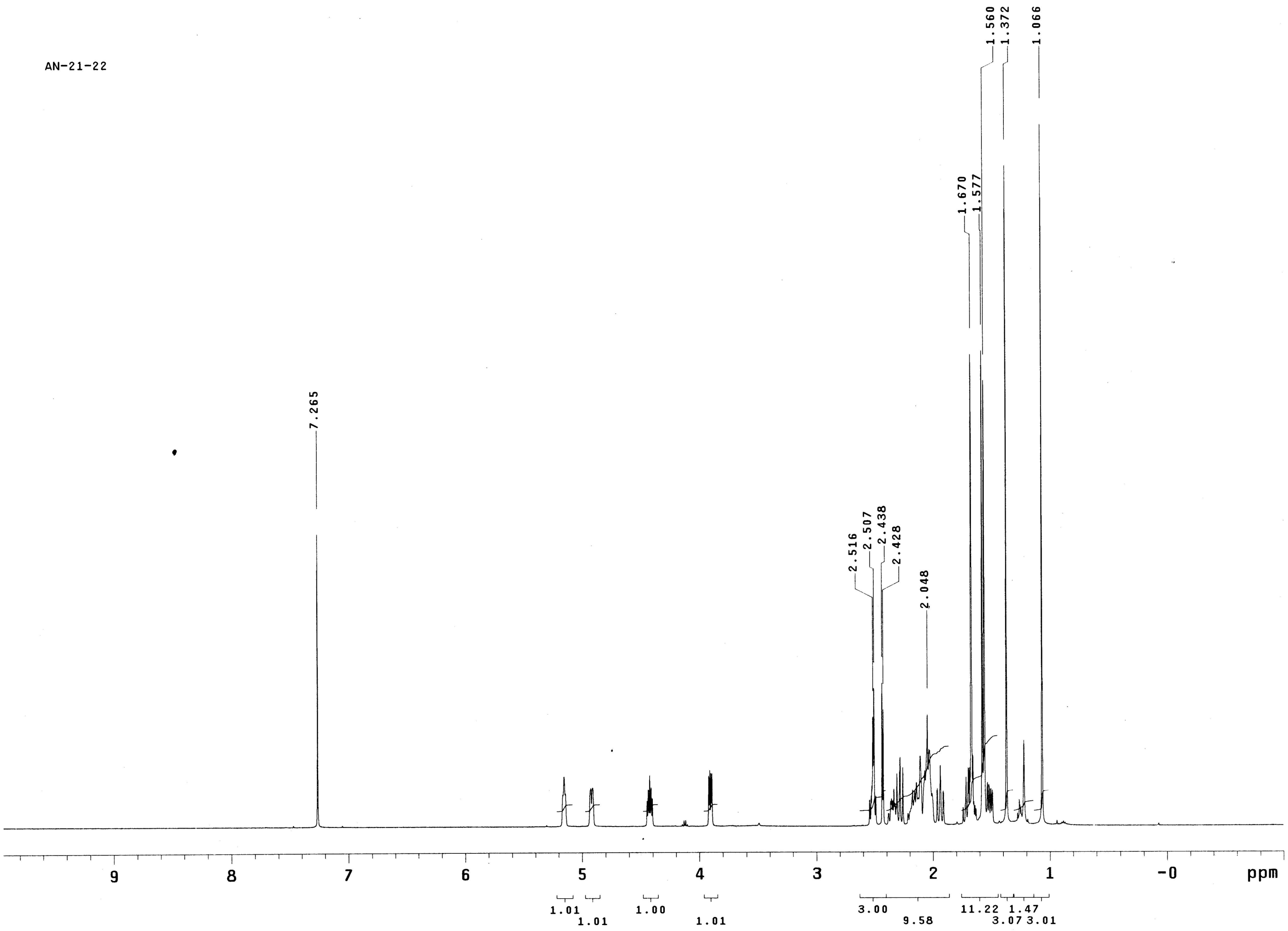

S4. ^{1}H NMR spectrum of **2** in $CDCl_3$ at 500 MHz.

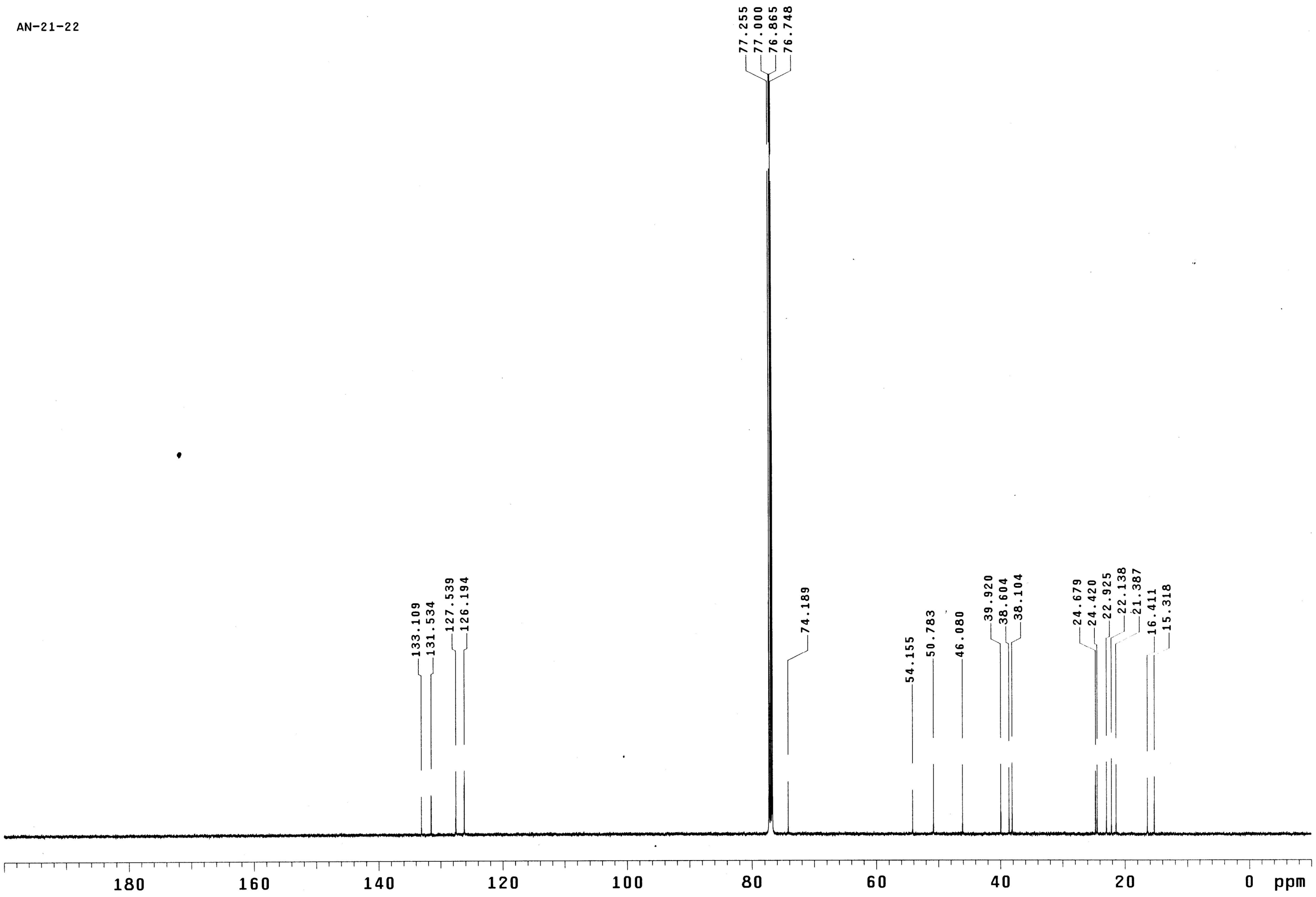

S5. ^{13}C NMR spectrum of **2** in $CDCl_3$ at 125 MHz.

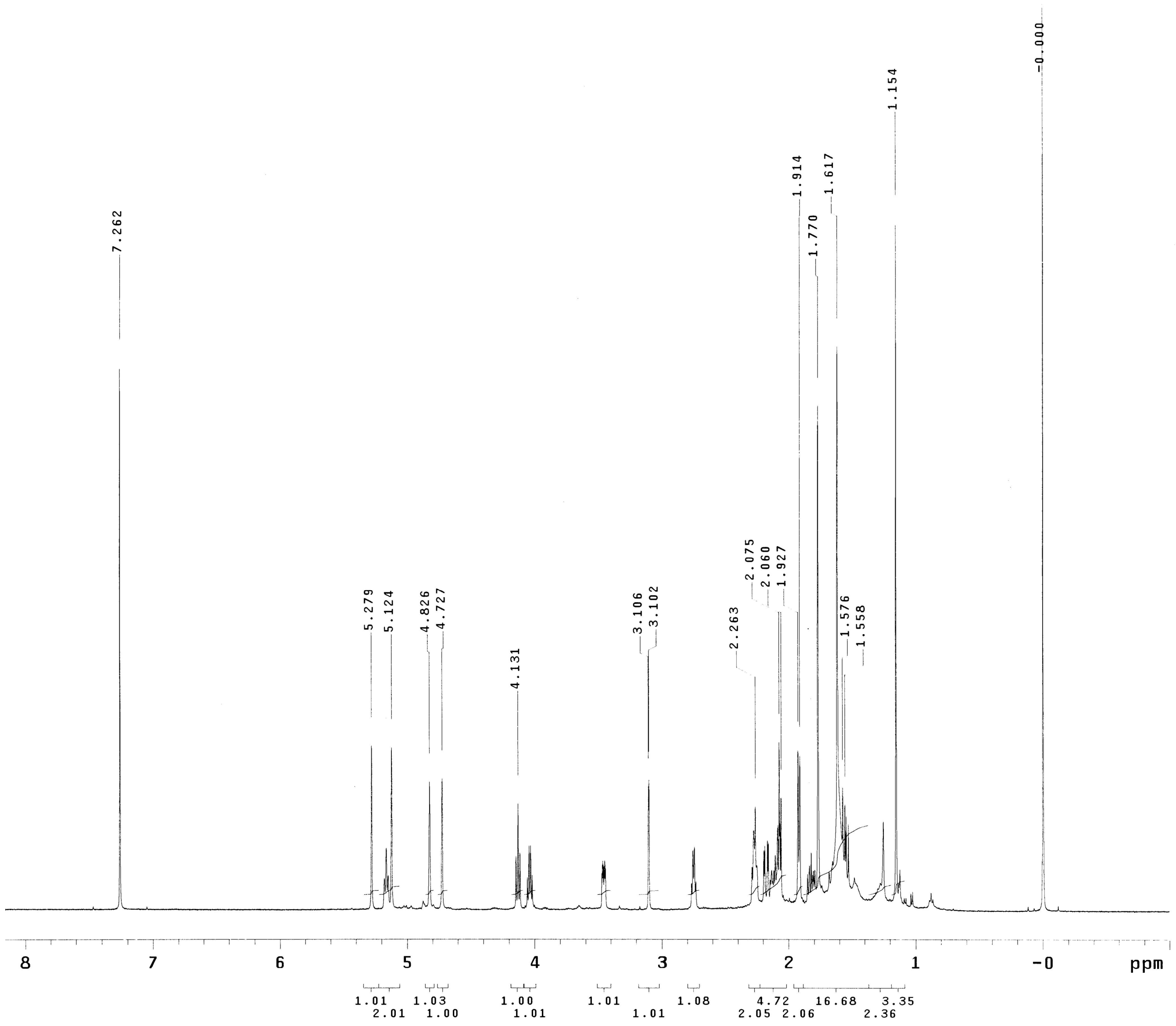

S6. ^{1}H NMR spectrum of **3** in $CDCl_3$ at 500 MHz.

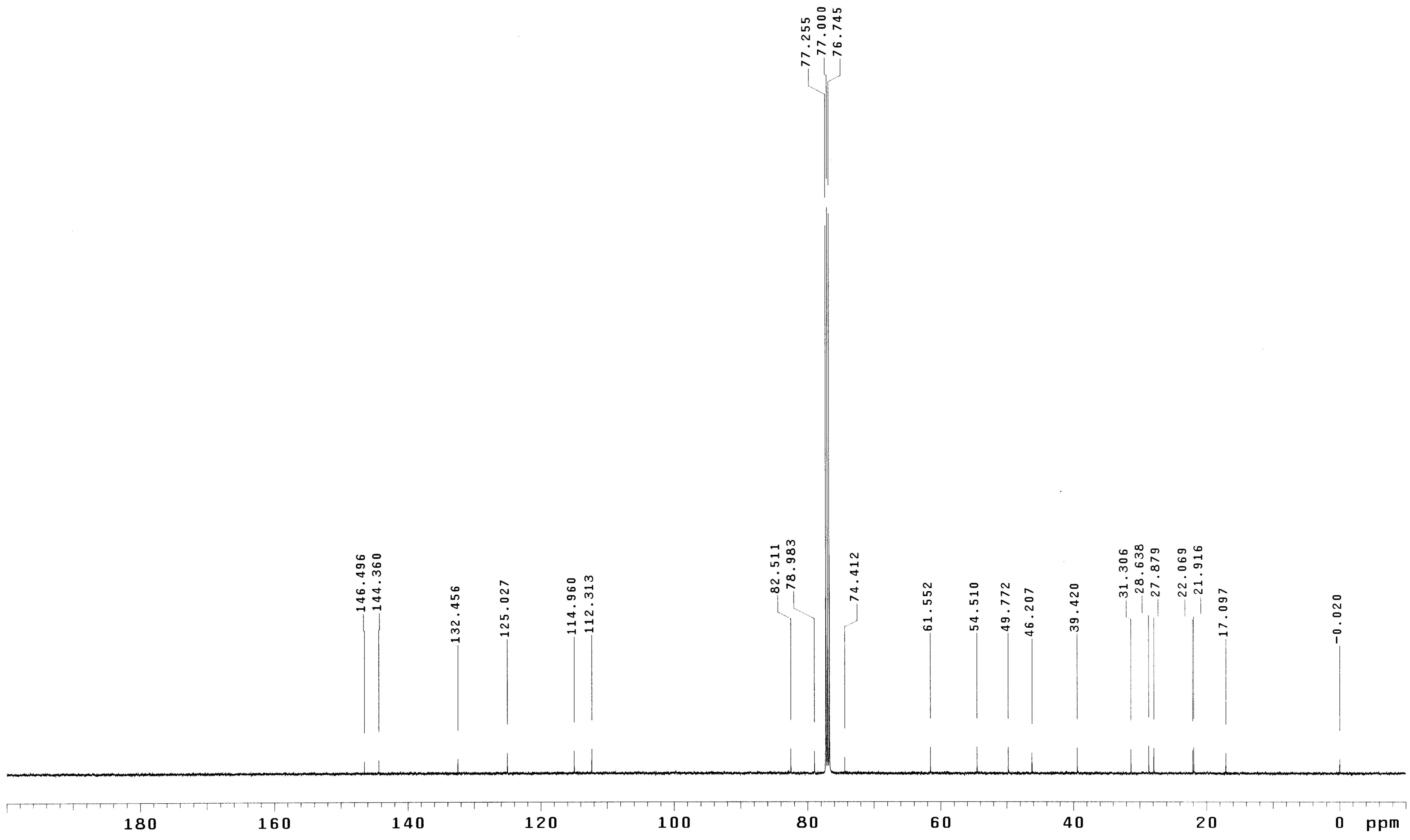

S7. ^{13}C NMR spectrum of **3** in $CDCl_3$ at 125 MHz.
